# Large Scale Molecular Studies of Pituitary Neuroendocrine Tumors: Novel Markers, Mechanisms and Translational Perspectives

**DOI:** 10.3390/cancers13061395

**Published:** 2021-03-19

**Authors:** Raitis Peculis, Helvijs Niedra, Vita Rovite

**Affiliations:** Latvian Biomedical Research and Study Centre, Ratsupites Str. 1-k1, LV-1067 Riga, Latvia; raitis@biomed.lu.lv (R.P.); helvijs.niedra@biomed.lu.lv (H.N.)

**Keywords:** pituitary neuroendocrine tumors, large scale molecular analysis, molecular markers

## Abstract

**Simple Summary:**

Pituitary neuroendocrine tumors are non-cancerous tumors of the pituitary gland, that may overproduce hormones leading to serious health conditions or due to tumor size cause chronic headache, vertigo or visual impairment. In recent years pituitary neuroendocrine tumors are studied with the latest molecular biology methods that simultaneously investigate a large number of factors to understand the mechanisms of how these tumors develop and how they could be diagnosed or treated. In this review article, we have studied literature reports, compiled information and described molecular factors that could affect the development and clinical characteristics of pituitary neuroendocrine tumors, discovered factors that overlap between several studies using large scale molecular analysis and interpreted the potential involvement of these factors in pituitary tumor development. Overall, this study provides a valuable resource for understanding the biology of pituitary neuroendocrine tumors.

**Abstract:**

Pituitary neuroendocrine tumors (PitNETs) are non-metastatic neoplasms of the pituitary, which overproduce hormones leading to systemic disorders, or tumor mass effects causing headaches, vertigo or visual impairment. Recently, PitNETs have been investigated in large scale (exome and genome) molecular analyses (transcriptome microarrays and sequencing), to uncover novel markers. We performed a literature analysis on these studies to summarize the research data and extrapolate overlapping gene candidates, biomarkers, and molecular mechanisms. We observed a tendency in samples with driver mutations (*GNAS*, *USP8*) to have a smaller overall mutational rate, suggesting driver-promoted tumorigenesis, potentially changing transcriptome profiles in tumors. However, direct links from drivers to signaling pathways altered in PitNETs (Notch, Wnt, TGF-β, and cell cycle regulators) require further investigation. Modern technologies have also identified circulating nucleic acids, and pinpointed these as novel PitNET markers, i.e., miR-143-3p, miR-16-5p, miR-145-5p, and let-7g-5p, therefore these molecules must be investigated in the future translational studies. Overall, large-scale molecular studies have provided key insight into the molecular mechanisms behind PitNET pathogenesis, highlighting previously reported molecular markers, bringing new candidates into the research field, and reapplying traditional perspectives to newly discovered molecular mechanisms.

## 1. Introduction

Pituitary neuroendocrine tumors (PitNETs) are glandular neoplasms of the anterior pituitary. The estimated prevalence of clinically significant tumors is approximately 1 per 1000 individuals [1], although evidence exists for underdiagnosed PitNETs in the general population [2,3]. Despite non-metastatic characteristics, PitNETs can increase or decrease the secretion one or more pituitary hormones, leading to increased morbidity and mortality [4,5,6,7,8]. Hormone-secreting PitNETs overproduce growth hormone (GH), adrenocorticotropic hormone (ACTH), prolactin (PRL), or rarely, hormones including follicle-stimulating (FSH), luteinizing (LH) or thyroid-stimulating hormone (TSH), leading to different systemic endocrine disorders, such as acromegaly, Cushing’s disease, and others. Untreated PitNETs, due to their size, may also exert mass effects, potentially promoting headaches, vertigo, and visual field defects (chiasma compression) [4,9].

From a medical perspective, some PitNETs are successfully managed with drugs targeting somatostatin (SSTR1-5) and dopamine (DRD) receptors. However, and with the exception of lactotroph PitNET, the overall efficacy of these drugs remains modest. Resistance to somatostatin analogs (SSA) has been reported in approximately 50% of patients with GH-producing PitNETs [8,10]. Treatment costs of €7000–9800 per patient/year [11] add a significant burden to healthcare budgets. Even more, the difference in cost between well-controlled somatotroph PitNET patients and PitNET patients with comorbidities, is up to €4500 per patient/year [12].

Although PitNET pathogenesis has been extensively studied, and specific oncogenes, tumor suppressors, and cell cycle regulators identified [13,14,15,16], the molecular factors underlying tumorigenesis, remission, and therapy responses remain unclear. Equally, it has also become evident that molecular mechanisms behind the development and response to sporadic PitNET treatment are heterogeneous in nature [17,18,19]. Accordingly, successful therapies require advanced molecular approaches to permit detailed tumor subtype classification, identification of novel drug targets, and monitoring markers to facilitate bespoke treatments.

Two major issues (among others) constrain the application of personalized treatment options: (1) limited factors defining variability in proliferation, hormonal activity, invasiveness, and response treatment, and (2) pituitary gland accessibility which limits tissue biopsy procedures, thereby hampering molecular biomarkers for observational or differential treatment purposes.

High throughput large-scale “omics” analyses have generated a spectrum of potential candidates for further analysis. Several studies have investigated both the inherited and somatic landscape of PitNET tumors [20,21,22,23], whereas others have reported transcriptome alterations potentially highlighting functional effects in PitNET cells [24,25,26,27] and others have investigated the potential impact of non-coding RNAs on PitNET pathogenesis [28,29,30]. In this review we analyzed the literature on large scale PitNET molecular studies to identify common candidates and markers for PitNET diagnostics, prognostics, and in-depth functional aspects of underlying molecular mechanisms.

### Review Terminology and Methodology

Peer reviewed study articles were taken from the National Center for Biotechnology Information database (https://pubmed.ncbi.nlm.nih.gov/, accessed on 5 October 2020). The primary goal of analyzing PitNET genomic and transcriptomic literature was to generate a comprehensive search of overlapping markers from studies conducted in previous years, therefore, no date limit was set for articles. In terms of microRNAs (miRNAs) and long non-coding (lncRNAs), we extracted pertinent research findings within the previous 3 years, as PitNET research had increased substantially in this period. Keywords for genomics articles included: pituitary adenoma genetics, PitNET genetics, familial pituitary adenoma, pituitary adenoma sequencing, and PitNET sequencing. For transcriptomics articles; pituitary adenoma transcriptome, PitNET transcriptome, pituitary adenoma RNA sequencing, PitNET RNA sequencing. For miRNA and lncRNA articles: pituitary adenoma, miRNA, circulating miRNA, and lncRNA. For the classification of different PitNET subtypes in the review tumors were categorized primarily based on the most recent terminology of the World Health Organization (WHO), where appropriate. In 2017, WHO published the Classification of Tumors of Endocrine Organs, which included a novel PitNET classification system [31]. The approach grouped PitNET tumors according to transcription factors specifying the cell lineage of endocrine cell origin. This classification is widely accepted in the literature, and is more predictive in terms of tumor prognosis [31,32,33]. We used this classification system where appropriate, as many review studies were published before 2017, and thus used a classification system based on clinical characteristics, e.g., PitNETs not secreting hormones were grouped as non-functional pituitary adenomas (NFPA). Therefore, for hormone-secreting tumors we used the following classification categories: somatotroph, lactotroph, and corticotroph PitNETs. For pre-2017 nonhormonal tumor studies, we used author classifications, because without adequate immunohistochemical data, it was not possible to group these tumors into new classification categories.

During the literature analysis, several issues were encountered. First, according to objectives, publications reported results using different filtering stages, ranging from almost unfiltered lists of genetic variants to a single or few molecular markers reinforced by experimental data. For the former, we performed additional filtering to exclude common population variants (variant has a dbSNP identification code and population frequency above 0.5%) or expression changes under a certain threshold (<1.5), whereas for the latter case we included high quality but limited scope knowledge from the particular study. Second, we encountered issues with the technical acquisition of data because not all studies had supplementary tables or data in easy-to-work-with formats. For example, some expression data were presented in a heatmap format without numerical backup tables, or numerical data described in the main text. Published data tables often required additional manual input in terms of data acquisition, and data access was limited for data presented in a portable document format (pdf). Notwithstanding these issues, data were extracted from texts and pdf tables and included. Third, we encountered comparison issues between study designs, timing, sample sizes, and technologies. Study comparisons were performed in a “single batch” i.e., after all data were extracted and formatted. We took into account limited knowledge at the time of the original study, and updated comparisons with contemporary knowledge and terminology. Our preference for explaining PitNET phenomena and properties focused on the latest studies with larger sample sizes, because they best reflected the current research environment. But we balanced this approach by reporting data from earlier studies and generating novel hypotheses by combining older data with contemporary technological data.

## 2. The Landscape of Genomic Alterations in PitNETs

Initial PitNET somatic genome investigations were published in the late 1990s. Comparative genomic hybridization (CGH) observations were conducted using chromosome number alterations and associated heterogeneity, and it was reported that the majority (>70%) of PitNETs were caused by chromosomal changes [34].

Despite technological advancements and advanced whole-genome investigations, the genetic background of PitNET remains largely uncharacterized. Genomic and genetic heterogeneity levels are high, therefore untangling the molecular causes behind individual PitNETs are challenging. PitNET tissue heterogeneity from surgery samples [35] and the lack of stable cell lines have further complicated this issue. PitNETs have monoclonal origin [36] but identification of tumor stem cells in PitNETs so far has been unsuccessful. Up to 95% of PitNET are sporadic, where somatic, mosaic, and familial low penetrance variations are the cause of neoplasms [37].

### 2.1. Familial PitNETs

Studies have shown that clinically significant PitNETs tend to occur in families [38] with syndromic disease, positive family history, childhood-onset PitNETs being the most important risk factors for disease prediction [37]. Therefore, initial research focused on PitNET genetic studies in familial disease settings. First, a gene responsible for multiple endocrine neoplasia type 1 (*MEN1*) was located, based on a study of three families [39]. Also, genetic changes, causing the Carney complex, which may produce PitNET in a patient, were ascribed to alterations in *PRKAR1A* [40] and *PRKARCB* [41]. Then, the cause of multiple endocrine neoplasia type 4 was elucidated using animal studies and *MEN1* mutation-negative patients and families, facilitating the discovery of *CDKN1B* in some familial PitNETs [42]. Previously described conditions often present themselves as PitNET but may develop other neoplasms in other tissue and organs. Multiple rarer disorders may manifest as PitNET, along with other neoplasms. These are: (1) so-called “3Pas” syndrome with mutations in a member of the succinate dehydrogenase gene group (*SDHx*) and/or MAX [43], (2) DICER1 syndrome with heterozygous germline variants in *DICER1* presenting as pituitary blastoma or corticotroph PitNET [44], (3) Lynch syndrome with germline *MSH2* [45] and *MLH1* variants detected [46]. Familial isolated pituitary adenomas (where only known neoplasm is PitNET) were extensively studied, with *AIP* cited as the molecular basis of disease [47,48]. Bridging familial and sporadic PitNET genetics (including MEN1), the X-linked acrogigantism (X-LAG) disorder was first characterized in both blood relatives and unrelated PitNET patients; with the genetic cause most likely a Xq26.3 genomic duplication, involving the candidate gene, *GPR101* [49].

### 2.2. Sporadic PitNET

Mutations in multiple genes (such as *GNAS*, *USP8*, *SF3B1*, and *MEN1*) have contributed to sporadic PitNETs in a specific or broad range of cases. This genetic heterogeneity confirms that from molecular standpoint PitNET is collection of disorders manifesting in tumors of the similar/same localization [50], yet attempts on PitNET genome-wide association study (GWAS) have been somewhat insightful. A cohort of 771 PitNET cases and 2788 controls in an East Asian population provided genetic evidence for the involvement of three separate chromosomal loci, 10p12.31, 10q21.1, and 13q12.13, indicating increased susceptibility to PitNET [51]. Although no specific genes were implicated, this discovery suggested inherited genetic variations were also part of tumor formation risk. Strikingly, no PitNET GWAS studies have been published since, probably due to a lack of sufficiently large cohorts, which often require cooperation between multiple research centers and countries. Following four genes with somatic variants have been identified as genetic causes in subgroups of certain PitNET hormonal types.

#### 2.2.1. GNAS

*GNAS* was the first gene identified in sporadic PitNET; several studies confirmed somatic *GNAS* variations in 15–40% of GH secreting PitNET patients [52,53,54]. *GNAS* is located at 20 q13.32 on the forward strand, and spans 71.47 kb [55]. The gene encodes a G protein α subunit, and mutations nullify or diminish GTPase activity of the α subunit, making it constitutively active. This activation, via increased cyclic adenosine monophosphate production (cAMP) and its dependent protein kinase A, which ensures the binding of CREB to PIT1 promoter regions, increases GH synthesis, and increases cell proliferation [37]. Two *GNAS* mutations associated with PitNET have been identified; p.R201C/S/H and p.Q227L/R [55,56,57]. The former accounts for the majority of cases, i.e., 89% (*n* = 8) [53] and 95% (*n* = 21) [58], while the latter was identified in one case in each study. The *GNAS* mutation, p.R201C was detected in other PitNET types: corticotropinoma [59] and in non-secreting PitNET [53]. *GNAS* mutation positive PitNETs usually have low (typically <12%) genomic disruptions (chromosome copy number alterations) [60] confirming *GNAS* tumorigenesis potential [61]. Copy number *GNAS* gains at chr 20q and the whole chromosome have been observed in PitNETs, whereas the absence of *GNAS* somatic mutations [60] are potential mechanism behind *GNAS* elevated activation in GH-secreting PitNETs [62].

#### 2.2.2. USP8

The first genetic success in PitNET etiology was the discovery of *USP8* (ubiquitin specific peptidase 8) somatic variants in patients with PitNET, secreting ACTH. *USP8* is located at 15q21.2 on the forward strand, spanning 90.04 kb [55] and encodes enzyme ubiquitin carboxyl-terminal hydrolase 8, which removes ubiquitin from other proteins, is capable of promoting receptor degradation, and is involved in protein sorting mechanism within a cell [63]. Somatic variations in *USP8* were identified in four out of ten corticotropinomas during exome sequencing [64]. These variations increased enzyme activity, and increased the levels of other proteins, including the epithelial growth factor (EGF) receptor [64]. Increased EGF signaling leads to increased cell proliferation [65]. The initial discovery that *USP8* somatic variations causecorticotroph PitNET has been replicated at least twice [66,67], including an East Asian population [66]. In the first validation study, 134 functioning and 11 silent corticotroph PitNETs were investigated; 48 of the functioning PitNETs harbored *USP8* variations, whereas none of silent PitNETs did [67]. Ma et al. observed that 67 of 107 PitNETs had *USP8* variations, suggesting a higher *USP8* somatic variation prevalence in this cohort [66]. Interestingly PitNETs with *USP8* somatic variations expressed high levels of pro-opiomelanocortin, somatostatin receptor 5 (SSTR5), and O-6-methylguanine-DNA methyltransferase (MGMT), and were more responsive to the somatostatin analog, pasireotide treatment [68]. The discovery of USP8 variations as causative mutations in a substantial fraction of functional corticotroph PitNETs facilitated the identification of a good drug therapy target. Moreover, USP8 inhibitors have been widely characterized for other human diseases, most notably, cancer [69,70].

#### 2.2.3. SF3B1

A recent study identified *SF3B1* p.R625H somatic variants in a subset of prolactin-secreting PitNETs. The discovery group of 21 prolactin-secreting PitNETs showed this as a recurrent variant in two tumors. The validation set of 227 tumors of the same type identified that 19.8% (*n* = 45) prolactin-secreting PitNETs contained this somatic variant. This study provided important insight into the functional mechanisms underpinning *SF3B1* somatic variants as promoters of aberrant splicing of estrogen-related receptor gamma, and associations with worse outcomes for patients [71].

#### 2.2.4. MEN1

Germline *MEN1* mutations were identified not only in familial cases but also in sporadic PitNET patients [22,25]. Particular PitNET hormonal subtype is not overrepresented, but young (<30 years) PitNET patients are more likely to have somatic *MEN1* mutation [72]. Also, the evidence suggested that both somatic variants [22] and single nucleotide polymorphisms (SNPs) were related to increased PitNET risk and associated with various characteristics [73].

### 2.3. Genome Changes in PitNETs

As expected, tumor somatic genome disruption range from negligible copy neutral loss of heterozygosity (LOH) to whole chromosome loss and chromosomal polyploidy. After the initial wave of PitNET CGH discoveries in the late 1990s and early 2000s subsided, PitNET genome studies received increased attention. Early CGH studies mostly had chromosome arm to whole chromosome-wide resolution [34,74,75,76,77], and concluded that chromosomal gains were twice as common as chromosomal losses, with some chromosomes experiencing more chromosomal gains, i.e., chromosomes X and 19 [34,74,75,76], whereas other chromosomes were more often partially or completely lost, i.e., 13, 11, and 18 [34,74,75,76,77]. Currently, whole-genome sequencing (WGS) and whole-exome sequencing (WES) are preferred genome characterization methods, either alone or coupled with array genotyping techniques [21,25], however, CGH is still used [60].

In terms of detection and reporting PitNET chromosomal alterations, reporting variations existed across studies; some did not report any variations [20,52] some reported whole chromosome changes [21,78] whereas others provided detailed insight [22,25,62]. Current studies/methods agreed with earlier CGH studies, suggesting chromosomal gains in PitNETs were on average twice as common and in twice as many tumors as chromosome losses [22,25,78,79]. The most common chromosome gains were of 7p and 19p arms, followed by 19q, 7q, and 8q arms, which occurred in >20% of tumors [25]. Previous studies indicated gains for these chromosomes, although at more modest levels [22,23,78,79]. For tumors with the most extreme amount of genomic material gain, tetraploidies in several chromosomal arms to whole chromosomes are common [25].

PitNET chromosome losses mostly occurred for 11 and the 22q arm [25] as previously indicated [22,23,78] although the affected tumor numbers were lower: i.e., 10–15%. Chromosome 11 was notable as it contains two well-known PitNET genes: *AIP* and *MEN1* s [55]. While more commonly studied in familial PitNETs, they also contained somatic variants in sporadic PitNET cases [22,80].

Usually, each individual PitNET has a dominant type of copy number alterations either gain or loss, with few tumors exhibiting both [25,62]. Based on copy number alterations, several studies have identified two distinct PitNET groups: low and high genomic disruption tumors [22,25,60,62] affecting <12–15%, and >24% of the genome, respectively. Low genomic disruption tumors experience a similar balance between chromosome gain and loss, while the high disruption group has more chromosomal gains [60]. It was discovered that GH-secreting PitNETs, with common *GNAS* driver mutations, had low genomic disruptions [60], suggesting PitNET tumors carrying driver mutations had a lower overall mutational rate when compared with tumors without a driver. This would mean that in tumor without drivers, somatic variants accumulate more gradually and eventually lead to tumorigenesis, while in PitNETs with driver mutations, the driver is the primary cause of tumor development.

In conclusion, with > 330 PitNET chromosomal alterations reported in the literature, it is clear that genomic disruptions are an important etiological aspect of PitNET biology, and are likely to determine individual tumor characteristics. However, tumor properties arising from gain or loss of whole chromosomes or arms (i.e., 7, 19, 8, 11, and 22) are yet to be determined. Currently, the only described fact states that response of GH to oral glucose loading and a dense tumor granulation is more prevalent in patients with high genome disruption in GH-secreting PitNETs [60].

### 2.4. WES, WGS Somatic Mutation Studies

The genome and exome sequencing era generated multiple studies investigating novel PitNET causing variations in genomic and somatic (tumor) DNA. The first small scale studies using <15 samples per study indicated low germline variation rates (<five per exome), and no reoccurring variants or genes were identified either within the study group or among multiple small-scale studies [20,21,78,79,81]. A 2016 study in 36 GH secreting PitNET cases, using exome sequencing, observed similar conclusions: a median of three protein-altering somatic variants per tumor (range: 0–13), recurrent *GNAS* variants, no other shared genetic variants between samplesor with samples of previous smaller scale research [21]. Yet, this GH PitNET study observed significantly increased somatic variations in G-protein coupled receptor (GPCR)/cAMP and calcium signaling pathway genes [52].

An East Asian population study used a systematic approach to sequencing seven clinical PitNET types: 20 non-functional PitNETs, 20 PRL-secreting PitNETs, 20 GH-secreting PitNETs, 20 ACTH-secreting PitNETs, 20 gonadotropin (including FSH and LH) secreting PitNETs, 10 TSH secreting PitNETs, and 15 mixed hormone PitNETs secreting at least two hormones [22]. Besides *USP8* (in ACTH secreting PitNETs), *GNAS* (in GH secreting and mixed secreting GH, and other hormone PitNETs), and *MEN1* (mixed PitNETs), six other genes (*KIF5A*, *GRB10*, *NR3C1*, *TRIP12*, *SP100*, and *IARS*) displayed somatic variations in at least two tumors, providing insight on other potential PitNET drivers. The authors also reported a median somatic variation rate per tumor exome at 3.3 (range 0–13) [22]. Another WES study using multiple PitNET types (*n* = 42) reported 12.5 non-synonymous variations per sample, and recurrent somatic variants in six genes; *ATAD3B*, *BHLHE22*, *KDM2B*, *OR5M1*, *TTN*,and *VPS13B*, although not the same base position [23]. In another study, exome analysis of recurrent PitNETs in the same patient indicated an increased load of somatic variants in the second tumor when compared with the first, suggesting an expansion of genetic changes as PitNETs progressed [81].

A recent large-scale study used a multi-“omics” approach to tackle PitNET origin and classification issues [25]. Although the main focus was transcriptome signatures, methylation and genomic rearrangements, somatic variants were also determined. In total, 6848 somatic variants were reported in 134 PitNET patients, combining exome data (*n* = 83 patients) and RNA sequencing (*n* = 51 patients) [25]. This study, and another [23], challenged the view that PitNETs harbored low amounts of somatic variants [20,52]. Changes in somatic variants per tumor could be attributed to improvements in sequencing technologies, data analysis, and methodological changes, thus removing the requirement for the validation of each somatic variant using other methods. However, increased somatic variant filtering and categorization could be provided across many studies. Of 6848 somatic variants [25], there were 5816 previously detected variants (position with corresponding snp138 db code) and 1032 novel somatic variants. 4264 unique SNP codes were among database variants, and the rest were repeating. 810 somatic variants had a population frequency >1%, and most likely represented undetected variants in normal DNA samples. Furthermore, 1868 of these somatic variants had a global population frequency >0.1% [25]. Of the novel somatic variants, 397 had no previous data in the gnomAD mutation database [82]. Also, novel somatic variants contained repeated somatic variants across two or more PitNETs in 20 positions in 15 genes; *DOCK8*, *ATN1*, *PABPC3*, *HLA-DRB5*, *AGAP4*, *SAMD14*, *USP8*, *VEZF1*, *FAM155A*, *EP400*, *ADAMTSL2*, *SEC16A*, *PCDHAC2*, *ALS2*, and *ENAH*. Several repeating positions were observed; *ADAMTSL2* at chromosome 9:136433542 had somatic variants in 17 PitNETs: five corticotroph, five gonadotroph, three somatotroph, and the rest were various. *USP8* had 14 somatic variants in corticotroph PitNETs, and three were different, but close proximity positions: chr15:50782647 (*n* = 7) chr15:50782640 (*n* = 7), chr15:50782639 (*n* = 3). *ATN1* had short indels in 15 tumors, and all were located in close proximity and varied in length from 3–12 bases. This was possibly a sequencing artifact of a trinucleotide repeat. Both *PABPC3* and *VEZF1* had somatic variants at the same position, in nine PitNETs of various types. Altogether, this study provided valuable insights into the somatic landscape of PitNETs and repeated somatic variants [25].

WES somatic variants were used to study cell cultures derived from PitNET surgical materials [35]. The data showed that two cell types typically found in cultures of primary surgery materials: pituispheres and mesenchymal stem cells were of different genetic backgrounds. Pituispheres represented PitNET tissue while mesenchymal stem cells represented normal or control genome composition, with no somatic variants characteristic to the tumor [35].

### 2.5. Recurrent Genes with Somatic Variants

As data on PitNET somatic mutations increases, the logical step is to compile a database and search for patterns and recurrences (Table S1). In this review, we identified four recurring genes (including *GNAS* and *USP8*) with somatic variants from WES and WGS PitNET studies, with less than 100 tumors. In addition to *GNAS* and *USP8*, these variants are located in *KLHL4* [20,35] and *RYR1* [21,35] genes. When a larger study with 125 PitNET patients was added [22], genes with somatic variants in at least two studies increase to 30 and adding novel somatic variants from the Neou et al.study [25], the increase is even more pronounced to 111 genes. Also, by adding all available studies, seven genes with somatic variants were identified in at least three studies (*ANKS3* [22,25,78], *GNAS*, *RUFY2* [21,22,25], *RYR1* [21,22,35], *SETD2*, *TTN* [22,23,25] and *USP8*). Also, repeating genes that in rare cases have been identified in the same study, are not readily discovered in other studies [22,79] (with the exception of established risk genes; *GNAS*, *USP8*, and *MEN1*). Only *KIF5A* bucked the trend, with somatic variants in two different tumors in the East Asian population [22] and another *KIF5A* variant was also discovered in a recent study [25].

## 3. Large-Scale Transcriptomics of PitNETs

PitNET transcriptome studies have been conducted over the previous 20 years, however, despite many discovered candidates, conclusive data on underlying genetic mechanisms of PitNET functionality have not been forthcoming. The transcriptome represents the part of the genome which is transcribed into RNA and translated into proteins therefore, transcriptome studies provide information on genomic regulation levels which may be translated to tumor properties, such as growth, invasiveness, and treatment responses. PitNET tumors, at large-scale transcriptional levels, have been studied using microarray [83,84,85] and RNA sequencing methods [25,26]. These reports compared the transcriptome in PitNET tumors from several study designs that give different angle on PitNET pathogenesis and functionality mechanisms: (1) PitNET vs. normal pituitary tissues [86,87,88], (2) invasive vs. non-invasive [24,84,89] or (3) comparison of expression between various PitNET types (somatotroph, lactotroph, corticotroph and other PitNETs [25,26]. Thus, we differentiated methodological approaches and study designs to better compare the literature data to determine overlapping candidates in each study design format (Table 1, Table 2, Tables S1 and S2).

### 3.1. Microarray-Based Approach

The first transcriptome-wide analysis of PitNETs was generated from microarray data in the early 2000s [90,91,92]. Microarray technologies evaluate the simultaneous expression of thousands of genes, providing information on transcriptional activities in biological samples.

Several transcriptome microarray studies investigated PitNET specific transcriptome hallmarks in comparison with normal pituitary tissue [88,92,93,94]. Evans et al. compared the transcriptome from different PitNET types (somatotroph, lactotroph, corticotroph PitNETs, and NFPA) with the normal pituitary gland. From 128 differentially expressed genes (DEGs), three tumorigenesis related candidates were selected for validation, and showed that the folate receptor (*FR*) was upregulated in NFPA, but downregulated in somatotroph and lactotroph PitNETs. C-mer proto-oncogene tyrosine kinase (*CMP-tk*) was upregulated in corticotroph PitNET, but downregulated in lactotroph PitNET, and ornithine decarboxylase (*ODC*) was upregulated in somatotroph, but downregulated in corticotroph PitNET [91].

A microarray study comparing 11 NFPA and eight normal pituitary tissues revealed 115 upregulated and 169 downregulated genes [92]. Subsequent validation analyses demonstrated significant upregulation of isocitrate dehydrogenase 1 (*IDH1*), paired-like homeodomain transcription factor (*PITX2*), and notch homolog (*NOTCH3*), whereas delta-like 1 homolog (Drosophila) (*DLK1*) was downregulated. These observations were further complemented by proteomic analyses, which demonstrated a 43% correspondence rate between proteome and expression changes. The authors concluded Wnt and Notch signaling were implicated in NFPA development [92].

In another microarray study, six lactotroph PitNETs and eight normal pituitary glands were investigated, and 726 DEGs identified [93]. Using validation studies, the authors demonstrated the significant upregulation of *TLE4*, *ANGPT1*, *DNAJB5*, and *NOTCH3*, and the downregulation of TGFBR3 in lactotroph PitNETs [93]. The authors proposed the involvement of Notch and Wnt signaling related factors, *NOTCH3*, *DLK1*, *HES1*, *ASCL1*, and *FDZ7* in prolactinoma development. Interestingly, the expression of several major histocompatibility complex related genes (*HLA-A*, *HLA-C*, *HLA-G*, *HLA-F*, and *B2M*) was altered, suggesting tumor immuno-surveillance issues in lactotroph PitNETs. This study also recorded changes in *ADAM28* expression [93] consistent with previous data [89].

A microarray study of 14 gonadotroph PitNETs versus nine normal pituitaries identified 1911 DEGs, including several involved in the p53 pathway [88]. The authors functionally characterized growth arrest and DNA damage-inducible β (*GADD45B*) factor, and demonstrated a role in PitNET tumorigenesis [88].

A recent computational study, using microarray data from data repositories, compared expression patterns of 14 gonadotroph PitNETs and nine normal pituitary tissues [94]. The authors identified 1220 DEGs, and having built a differentially co-expressed gene network, they suggested *DLK1*, cyclin-dependent kinase inhibitor 2A (*CDKN2A*), integrin alpha 4 (*ITGA4*), palmitoylated membrane protein 2 (*MPP2*), ArfGAP with SH3 domain, and ankyrin repeat, and PH domain 2 (*ASAP2*) were involved in gonadotroph PitNET development and pathogenesis [94].

Several studies also compared microarray transcriptome characteristics between different PitNET types, to identify markers for each subtype. Expression microarray data of five pooled PitNET samples from each subtype (somatotroph, lactotroph, corticotroph PitNETs, and NFPA) identified 3906 genes, with 351 expressed sequence tags differentially expressed in all PitNET types when compared with normal pituitary tissue. The validation of three oncogenesis promoting candidates indicated that lysosomal-associated protein transmembrane-4-b (*LAPTM4B*) was upregulated in corticotroph PitNETs and NFPA, Bcl-2-associated athanogene (*BAG1*) was upregulated in somatotroph, lactotroph PitNETs, and NFPA, and cyclin-dependent kinase inhibitor p18 was downregulated in corticotroph PitNETs [90].

Hu et al. performed an extensive computational study comparing microarray profiles of 160 tumors and normal tissues, including 52 corticotrophs, 38 somatotrophs, nine lactotroph PitNETs, 40 NFPAs, and 21 normal pituitary glands [95], combining microarray data from [84,85,88,96,97]. Tumor subtype-specific DEGs were identified; 22 for somatotrophs, 1081 for lactotrophs, and 437 for NFPAs, whereas 217 DEGs were common to all PitNET types. The authors postulated alterations in metabolic processes i.e., fatty acids, amino acids, nucleotides, carbohydrates, and other metabolic pathways [95], however, samples from various studies and ethnic background may have affected this data.

Other reports also identified novel markers implicated in PitNET growth by dividing tumors into subgroups according to invasiveness characteristics [89]. A study of 10 lactotroph PitNET tissues (three non-invasive, four invasive, and three aggressive invasive) and normal pituitary glands, suggested 33 DEGs discriminated invasiveness in each lactotroph PitNET type. DEG functions were related to six functional classes: (1) invasiveness; *CRMP1*, *SLITRK3*, and *DCAMKL3*, (2) proliferation; *CENPE*, *PTTG*, *CCNB1*, *KIF13B*, *TRIB3*, *CENPE*, *HIST1H4H*, and *MTB*, (3) transcription regulation; *ZNF568*, *KIAA1729*, *PHF12*, and *PHF19*, (4) pituitary development; *PITX1*, (5) extracellular matrix regulation; *ADAMTS6*, and (6) cell cycle *ASK*, *RACGAP1*, *CENPE*, and *AURKB*. Validation studies confirmed the relationship of invasiveness associated gene expression patterns and histological markers (Ki-67, mitosis rate) [89].

Another study investigated eight invasive and eight noninvasive PitNETs, and discovered 194 DEGs were shared between groups. Pathways involving leukocyte transendothelial migration, cell adhesion, and adherens junctions were linked to increased invasiveness in PitNETs [98]. In experimental studies, three invasive and four non-invasive NFPAs were compared and 1160 DEGs identified; the most relevant pathways included cellular growth and proliferation, cellular movement, and cellular development. Validation of two candidates indicated that chromogranin A (*CHGA*) was downregulated, but clusterin (*CLU*) was upregulated in invasive NFPA. Importantly, this was also confirmed at the protein level [97]. Furthermore, Chen et al. investigated invasiveness in three invasive and four noninvasive NFPAs, identifying 1472 shared DEGs between groups. The authors identified several candidates potentially related to invasiveness phenotypes; *CAT*, *CLU*, *CHGA*, *EZR*, *KRT8*, *LIMA1*, *SH3GLB2*, and *SLC2A1*. Using functional studies, *EZR* knockdown decreased cellular invasiveness properties [99].

Corticotroph PitNET invasiveness was assessed in three tumor groups: four noninvasive micro- corticotroph PitNETs, five noninvasive macro-corticotroph PitNETs, and three invasive macro- corticotroph PitNETs [84]. The authors discovered 168 DEGs shared between invasive and non-invasive corticotroph PitNETs, identifying TGF-β and G protein signaling, DNA damage responses and cell cycle control in the invasiveness phenotype. Subsequently, *CCND2*, *ZNF676*, and *DAPK1* were validated with altered expression levels in invasive corticotroph PitNETs [84]. A more extensive study using 40 mostly gonadotroph type PitNETs, investigated 22 invasive and 18 non-invasive tumors [83]. These authors discovered 346 DEGs when comparing both groups, and identified genes for tumorigenesis, cell movement, and transcriptional regulation functions. The validation of four of 35 selected candidates was successful; *IGFBP5*, *MYO5A*, *FLT3*, and *NFE2L1* [83]. Levka et al. performed microarray analysis in tissue with low E-cadherin (*CDH1*) expression (eight somatotroph PitNETs) and high *CDH1* expression (eight somatotroph PitNETs) [85]. They identified 150 DEGs of which 20 were downregulated and nine upregulated, with roles in epithelial-mesenchymal transition (EMT). Extended functional analyses demonstrated the potential involvement of Epithelial Splicing Regulatory Protein 1 (*ESRP1*) in EMT regulation in somatotroph PitNETs [85].

Potential recurrence markers for PitNETs were assessed in another microarray analysis on four recurrent, and seven primary non-secretory PitNETs [100]. Here, the authors identified 70 DEGs, the most significant being; *CHL1*, *P2RY12*, *ERAP2*, *LPAR5*, and *CHAD*. The cell adhesion molecule L1 like gene (*CHL1*) was speculated to contribute to tumor recurrence [100].

Another microarray study of 40 corticotroph PitNETs investigated sample clustering in tumors based on expression profiles, and divided tumors into three groups correlated with macro-tumor percentage in the group, extrasellar extension, and age [96]. The authors suggested corticotroph PitNETs exhibited transcriptome profiles related to neuroendocrine cell functionality, involving biosynthesis enrichment, RNA processing, metabolic, membrane, and vesicular pathways, manifesting in transcriptional profile related correlation with clinical patterns [96].

Microarray studies investigated specific candidate gene differences in *AIP* mutation-positive somatotroph PitNETs (six *AIP* germline variant somatotroph PitNETs, seven wild-type *AIP* variant somatotroph PitNETs, seven NFPAs, and five normal pituitaries) [101]. No significant differences were observed between somatotroph PitNET with and without *AIP* germline mutations, however, alterations in β tubulin proteins (*TUBB2B*, *TUBB6B*) between the groups were detected, and increased *NME1* and *HSPA9* expression was demonstrated in a group with a germline *AIP* mutation [101].Another microarray study of *AIP* mutation positive tumors identified significant alterations in *EMT* when compared with sporadic tumors, and five specific markers, *CDH1*, *ESRP1*, *EPCAM*, *PERP*, and *ZEB1* exhibited significant changes also in validation set [102]. These authors observed *CCL5* upregulation in tumors bearing *AIP* mutations. Functional studies revealed that *CCL5* increased macrophage infiltration in *AIP* mutation positive tumors, suggesting a putative role in of tumor microenvironment related to PitNET aggressiveness [102].

Candidate genes/pathways identified in microarray studies rarely overlapped, however, several reports identified Notch, Wnt, [92,93], and TGF-β pathways [84,93], and also cell cycle regulators [94,95] as overlapping candidate regulatory mechanisms. Thus, signaling pathway heterogeneity may have depended on experimental design. Different studies used different processing for biological samples; some used paraffin-embedded tissues, others, frozen samples, whereas microarrays were variably distinct using different probe quantities, i.e., 7000 to >20,000. Similarly, reports had distinct study designs, different sample grouping strategies based on clinical or molecular parameters, and different data analysis approaches. Another factor affecting heterogeneity was tumor subtyping, as some reports used tumor pathological classification, while other used clinical characteristics. These comparisons and overlapping candidates are shown (Table 1, Table 2, Tables S1 and S2).

### 3.2. Whole Transcriptome Sequencing and Pangenomic Classification of PitNETs

Whole transcriptome sequencing (RNAseq) is a high throughput method which in principle, overcomes limitations of the microarray-based approach, in particular for probe set variability, which could affect crucial transcript discovery. As for microarray-based transcriptomic studies, the first RNAseq reports included small numbers of PitNET samples [24,27,86,103]. The RNAseq analysis of four null cell PitNETs and four silent subtype III PitNETs which were more clinically aggressive, demonstrated altered gene expression in tumor microenvironment and immuno-surveillance regulation, e.g., arginase type II (*ARG2*), semaphorin-3A (*SEMA3A*), and some *HLA* genes [103]. Of note, the p53 signaling pathway was significantly upregulated [103]. Another RNAseq study on 13 corticotroph PitNETs and five normal pituitary tissues identified 122 DEGs [86]. Secreted frizzled-related protein 2 (*SFRP2*) was significantly decreased and later confirmed in functional studies, suggesting *SFRP2* had tumor suppressor properties downregulating Wnt pathway. Other significantly enriched pathways included TGF-β and p53 signaling, cell cycle regulation, and other pathways [86]. RNAseq analyses in subgroups of fast and slow-growing gonadotroph PitNETs, based on tumor volume doubling times, identified 350 DEGs [27]. Upon validation of 40 candidates, 11 demonstrated correlations with tumor volume doubling times, which included six EMT pathway members [27]. Kim et al. investigated three noninvasive and 11 invasive NFPAs, and identified 700 DEGs between groups [24]. Several immunity-related genes (*IGKC*, *C1S*, *C1R*, and *IFITM1*) and *TGF*-β pathway genes (*TGFRB2* and *TGFB*) were downregulated. It was speculated that claudin-9 (*CLDN9*), insulin-like growth factor-binding protein 5 (*IGFBP5*), death-associated protein kinase 1 (*DAPK1*), and tissue inhibitor of metalloproteinase-3 (*TIMP3*) were potential contributors to the invasive phenotype [24].

An RNAseq investigation analyzing both mRNA and lncRNA (lncRNA data are described below) levels demonstrated 1015 significantly altered mRNAs between eleven gonadotroph PitNETs and five normal pituitary tissues [87]. Pathway enrichment analysis demonstrated that transcription regulation, cell metabolism, and proliferation functions were involved in gonadotroph PitNETs when compared with control pituitaries. Ingenuity pathway analysis also implicated mTOR, EIF2, cellular junctions, integrins, and other pathways [87].

Interestingly, Salomon et al. alongside RNAseq has performed exome sequencing and methylation analysis, all were assessed to adjust different “omics” landscape in 48 tumor tissues’ subtypes of somatotroph, corticotroph PitNETs, and endocrine-inactive PitNETs [26]. Transcriptomic profiles clustered together based on PitNET subtypes, which was similarly observed for methylation profiles. In somatotroph PitNET, elevated *SSTR5*, *GH1*, and *GH2* expression was observed, and corticotroph PitNETs had increased proopiomelanocortin (*POMC*) expression. Of note, no specific transcriptional changes were detected in tumors bearing *USP8* or *GNAS* mutations [26].

This study also investigated PitNET transcriptome patterns based on SSA treatment status before surgery. The data indicated tumors with preoperative therapy exhibited decreased Ki67 expression, and increased *MUC1* and *CD40* expression, indicative of tumor development and cell differentiation [26]. In a recent study assessing six somatotropinomas undergoing SSA/DA treatment and six without therapy, before tumor resection, tumors exhibited distinct transcriptome patterns based on treatment status before surgery [104]. Several tumorigenesis related factors were downregulated in tumor tissue upon SSA/DA therapy; *MUC16*, *MACC1*, and *GRHL2* [104].

Another multi “omics” study which investigated 134 major PitNET subtype tumors, provided novel insights for PitNET biology [25]. Firstly, the study undermined the 2017 WHO of PitNET classification system. The authors demonstrated that transcription marker, *SF-1* expression was not restricted to gonadotroph PitNETs, but was highly detectable in somatotroph PitNETs, and that corticotroph and gonadotroph coexpression patterns were detected in silent corticotroph PitNETs. Secondly, authors observed similar major tumor subtype clustering based on “omics” data as previously reported [26], however, clear differences in *USP8* and *GNAS* expression patterns in mutated tumors were identified when compared with wild-type [25], but not indicated by Salomon et al. [26].

Another study used CIBERSORTx computational analysis to depict immune cell abundance in mixed cell populations and using RNA-seq data from previously discussed publication [25]. The authors discovered that M2 macrophages are predominantly found immune cell type in PitNETs, additionally each subtype PitNET tissue have specific immune cell profiles [105]. This “omics” study used available online data repositories to answer novel and relevant research questions, indicating the importance of data reuse for PitNET biology.

In another study, RNA-seq was used to investigate 115 PitNET samples to analyze immune cell abundance [106]. These authors identified increased B and CD8+ T cell numbers in somatotroph PitNETs, and tumor type specific immune profiles [106] similar to [105]. This study highlighted the potential use of cancer vaccines and checkpoint targeting treatments for PitNET management [106].

Novel approaches to PitNET characterization are also being pursued. XCell deconvolution digitally dissects tumor microenvironment using in silico approaches. A recent study used this method to analyze PitNET microarray data [107], where macrophage infiltration was more than three times higher in PitNETs when compared with the normal pituitary. Importantly, these observations were corroborated by immunohistochemistry [107], indicating excellent method feasibility in characterizing PitNET immunoprofiles.

Pure computational analyses on NFPA “omics” data from transcriptomics and proteomics studies have also extrapolated vital signaling pathways involved in NFPA pathogenesis, including; PI3K/AKT, mTOR, Wnt, and ERK/MAPK pathways [108].

Interesting questions have arisen from these RNA sequencing studies, i.e., what is the impact of potential *USP8* or *GNAS* driver mutations on tumor transcriptome profiles [25,26], and how does the expression of distinct PitNET cell lineage transcription factors affect other tumor lineage types [25]. Therefore, independent data sets with comparable sample sizes are required to answer these questions.

### 3.3. Overlapping Transcriptome Markers

As previously described, different large-scale PitNET transcriptome studies used different methodologies and study designs to group samples [25,26,27,96]. Thus, for this review, we divided these studies according to design, and investigated potential overlapping gene candidates between studies (Table 1, Table 2, Tables S1 and S2).

A limitation of this combinatorial approach was that for some publications, study designs were not directly comparable, therefore we did not include these reports in the search for overlapping candidates. For example, Neou et al. classified PitNETs according to the WHO classification system [25], but in previous reports, PitNETs were mostly grouped according to clinical characteristics [26,86,95].

Nevertheless, we identified overlapping transcriptome factors in more than three publications. For study designs which compared different PitNET types with normal pituitary tissue, often DEGs were related to hormone secretion, i.e., *POMC*, *GH1*, *GH2*, and *CSHL1* (Table 2) [87,88,92,93,95]. We also observed overlapping factors, such as *DLK1* related to Notch signaling, *NNAT* involved in pituitary development [92,93,95], *GAL* related to growth hormone release [87,88,92,95], *NPTX2* involved in cell plasticity [92,93,95], and *PMAIP1* which promotes apoptosis [86,92,95,109]. The functions of these factors indicated their potential involvement in PitNET development and clinical manifestation. In other studies, the chemokine, *CCL2* was also described as a potential regulator of the PitNET microenvironment [107,110]. Notably, *CCL2* was one of the main chemokine secreted from PitNET surgery derived primary tumor tissue [107]. In several studies, the PitNET microenvironment was investigated, and several factors; *CCL5*, *CXCL8*, *CXCL12*, *CXCR4*, and *CXCR7*, and other are discussed that potentially influence aggressiveness and clinical properties of PitNETs [26,102,106,111,112,113,114]. However, comparing different transcriptome analysis in our study we did not depicted specific up- or downregulation of indicative tumor microenvironment markers that overlap between several studies. For other overlapping candidates, the involvement of *PON3* and *F3* in PitNET development is unclear.

In studies investigating PitNET growth and invasiveness, we observed one candidate which overlapped in three studies; *TRIM36* (Table 1) [27,85,89]. *TRIM36* is involved in the ubiquitin pathway and the proteasomal degradation of target proteins [109]. No reports have functionally investigated *TRIM36* with respect to PitNET, therefore its role PitNET invasiveness could be further studied.

Many studies have reported the involvement of cell cycle regulators; *CDKN1B*, *CDKN2A*, *CDKN1C*, and others (Table 1 and Table 2), however, we observed no particular factors which overlapped in more than two of the included reports. This could indicate that cell cycle regulation is an important part of PitNET pathogenesis, but the heterogeneity of each tumor leads to the different disturbances in these pathways.

## 4. Micro RNAs in PitNET Pathogenesis

Micro RNAs (miRNAs) are a class of small non-coding RNA expressed in all eukaryotes, with an average length of 22 nucleotides (nt), but varying between 21–25 nt [115,116]. In recent decades, miRNAs have become synonymous as post-transcriptional regulation factors in humans; according to Friedman et al.: “60% of human protein-coding genes are conserved targets for miRNAs” [117]. The first studies were published in 2002, and indicated miRNAs were associated with tumor growth; authors observed that miR-15a and miR-16-1 [located on chromosome 13q14 which also contains a well-known tumor suppressor gene retinoblastoma, (*RB*)] were inversely correlated with leukemia development [118,119]. Interestingly, these miRNAs were also associated with PitNETs; decreased miR-15a and miR-16-1 expression correlated with increased GH mass and prolactin-secreting PitNETs [120].

According to PubMed, 2019 had the highest number of research articles on PitNET related miRNAs. These studies identified several novel miRNAs which require further investigation as potential biomarkers and therapeutic targets for PitNET. A recent Chinese study, using next generation sequencing (NGS) and qPCR validation, identified 10 differentially expressed miRNAs between PitNET and normal pituitary autopsy samples [30]. Of interest, miR-34c-5p, miR-338-5p, and miR-378, which are previously known tumor suppressive miRNAs, were also significantly downregulated in lactotroph PitNETs [30]. These authors commented that miR-34c-5p had two tumor suppressors; *CDH1* and *TGFBR2*, and one oncogene, *ERBB2*, as putative targets [30]. However, these were not the only miR-34c-5p targets, as it was previously reported miR-34c-5p interacts with AREG glycoprotein mRNA. AREG serves as a ligand for the EGFR and is overexpressed in ovarian cancer [121]. According to The Human Protein Atlas database (v 20.0), *AREG* is also expressed in the pituitary gland [122]. Other studies indicated that miR-338-5p interacted with the 3’ untranslated region of the *EGFR* oncogene and *PIK3C3*, a candidate tumor suppressor, however, other studies questioned this *EGFR* interaction [123,124]. The downregulation of miR-378 was previously observed in adrenal adenomas, with a proposed target gene, *MTDH*. *MTDH* is involved in the EMT, and its upregulation correlated with more aggressive phenotypes in various tumors, including PitNETs.

The most common characteristic of invasive PitNETs is their rapid expansion to nearby structures [125]. A recent NGS study compared miRNA expression profiles [27,126] between invasive and non-invasive PitNETs and revealed 55 differentially expressed miRNAs. miR-665 was the most upregulated miRNA in invasive PitNETs [127]. Overexpressed miR-665 was first identified in hepatocellular carcinoma, and appeared to influences tumor aggressiveness by interacting with *PTPRB*, which affected the YAP-Hippo pathway [127,128]. The most downregulated miRNA was miR-149-3p [127], which was interesting, because another study reported that miR-149-3p was significantly downregulated in NFPAs when compared with hormone-secreting PitNETs [129]. Study authors reported that 10 of 12 NFPA cases exhibited cavernous sinus invasion, while five of 11 hormonally active PitNETs had no cavernous sinus-invasion, suggesting miR-149-3p served as an invasion marker for NFPA. This observation was further supported by evidence that miR-149-3p targeted *SMAD3*, a gene which is involved in the TGF-β pathway, and reportedly upregulated in invasive NFPA [129,130,131].

Another important topic is the association of miRNAs with therapeutics resistance. For prolactinomas, dopamine agonists (cabergoline, bromocriptine) (DA) are widely used treatments. In most cases, these agonists successfully normalize blood prolactin levels, however, resistant cases are prevalent: 20–30% for bromocriptine and 10% for cabergoline [132]. Wu et al. investigated bromocriptine resistant and sensitive PitNETs using NGS and PCR validation [133]. They identified nine upregulated and three downregulated miRNAs in bromocriptine resistant tumors. Gene ontology and Kyoto Encyclopedia of Genes and Genomes analyses identified that two upregulated miRNAs (miR-93 and miR-17) had potential roles in resistance formation, as they appeared to regulate 94 target genes, including *CDKN1A* a cell cycle regulator associated with chemosensitivity in some cancers [133,134,135]. In vitro miR-93 investigations provided further evidence that *CDKN1A* was a direct target of miR-93; however, miR-93 overexpression only slightly increased resistance to bromocriptine (7–8%), and a similar effect was observed for mir-17. This suggested that the remaining 10 miRNAs were similarly implicated in bromocriptine resistance [133].

A recent study explored miR-93 associations with cabergoline resistance [136]. In vitro assays confirmed that miR-93 overexpression increased GH3 cell resistance to cabergoline treatment, by reducing cabergoline-induced autophagy. This effect was explained by the miR-93 mediated regulation of *ATG7* expression; *ATG7* was previously associated with cabergoline resistance [136,137]. Overall, these studies suggested miR-93 predicted dopamine agonist therapy resistance, therefore its clinical applications should be investigated.

### Circulating miRNAs

Over the past decade, the role of circulating miRNAs, particularly vesicle bound miRNAs, has been extensively studied as liquid biopsy-derived biomarkers in cancer diagnostics [138]. These extracellular miRNAs are located in bio-fluid samples such as plasma, with normal tissue and tumor origins. Current evidence has shown that circulating miRNAs act as both diagnostic and prognostic biomarkers for various cancers [138]. The first study was published in 2012, where three miRNAs related to tumor progression: miR-21, miR-128, and miR-342-3p were investigated in plasma [139]. A quantitative (q)PCR approach in gliomas, meningiomas, and PitNETs was used. While these miRNAs were significantly dysregulated in plasma of glioma patients, this was not the case for PitNETs [139]. The next circulating miRNA study was published by Nemeth et al. who used a deep NGS and qPCR validation approach, and reported that miR-143-3p downregulation was directly linked to the resection of FSH/LH-secreting PitNETs, suggesting its utility as a plasma biomarker in monitoring relapse risk post-surgery [140].

For Cushing’s syndrome (CS), approximately 20–25% of cases have an ectopic (non-ACTH secreting PitNET) origin. Even though PitNETs are responsible for 75–80% of cases in clinical practice, distinguishing CS with PitNET origins from ectopic origins is challenging [141]. Bilateral inferior petrosal sinus sampling provides highly sensitive and specific results for diagnosing ACTH-secreting PitNETs, but the procedure is invasive, costly, and requires trained specialists [142]. For this reason, Belaya et al. evaluated which circulating plasma miRNAs could serve as markers to distinguish CS caused by ACTH-secreting PitNET (CD) versus CS of an ectopic origin. They enrolled 41 patients (28 with ACTH-secreting PitNET and 13 with ectopic CS. MiRNA quantification and differential expression analyses identified three upregulated miRNAs in patients with ACTH-secreting PitNET: miR-16-5p, miR-145-5p, and let-7g-5p. MiR-16-5p and miR-7g-5p were particularly interesting as they were also upregulated in patients with PitNET when compared with healthy controls, further hinting that the source of these miRNAs was the tumor itself [143].

Studies on circulating plasma miRNAs in PitNET patients are scarce, with only four proposed candidate markers identified so far: miR-143-3p, miR-16-5p, miR-145-5p, and let-7g-5p [140,143]. By comparing these findings to miRNA studies in tissues of PitNETs and other tumors the expression values of these miRNAs in plasma are inverse to findings in tissue studies (Table 3). For example, plasma miR-143-3p was upregulated before surgery, when compared with plasma three months after transsphenoidal PitNET resection [140]. In several tumor tissue studies, miR-143-3p was significantly downregulated and acted as a tumor suppressor by silencing genes which promoted cell proliferation and invasion, i.e., *COX-2* in gastric cancer, *FOSL2* in osteosarcoma, and *KRAS* in PitNET [144,145,146]. For miR-145-5p (upregulated in plasma) [143], an in vitro study demonstrated it increased prolactin-secreting PitNET cell susceptibility to bromocriptine therapy, was downregulated in therapy-resistant tumors, and targeted *TPT1*, a cell survival-promoting factor with a crucial role in the p53 pathway [147,148]. Several tumor studies reported miR-16-5p (upregulated in plasma) was downregulated, suggesting this miRNA most likely functioned as a tumor-suppressive miRNA. It achieved this effect by interacting with *ATK3* (NF-κB pathway) and *VEGFA* in breast cancer, and *SMAD3* in chordoma and osteosarcoma [149,150,151,152]. As for let-7g-5p, it was reportedly downregulated and acted as a tumor-suppressive miRNA in ovarian and gastric cancers [153,154]. Thus, circulating miRNAs which are upregulated in plasma, may be downregulated in PitNET (Table 3). Perhaps, a mechanism which permits tumor proliferation involves an excessive dumping of tumor-suppressive miRNAs. Future studies should investigate both tumor tissue and plasma miRNAs from the same PitNET sample set to further evaluate this claim.

## 5. Regulatory Effects of Long Non-Coding RNA in PitNETs

Long non-coding RNAs (lncRNAs) are another class of regulatory RNAs, >200 nt. They have important roles in embryogenesis and cellular processes, involving cell cycle regulation and apoptosis [155]. LncRNAs are transcribed mostly by RNA Polymerase II, and unlike miRNAs, the mechanism through which lncRNAs mediate their regulatory effects are more diversified. LncRNAs can silence multiple genes by recruiting chromatin remodeling factors [156], and interact with miRNAs to act as sponges to downregulate their effects on respective target mRNAs [157]. Thanks to their gene expression regulatory capabilities, lncRNAs are linked to the pathogenesis and progression of various tumors, including PitNETs. Xing et al. compared tumor samples from NFPA patients against normal pituitary autopsy samples [29]. Using a microarray approach authors identified 113 differentially expressed lncRNAs in NFPAs. 10 lncRNAs (fourdownregulated and sixupregulated) were randomly selected for validation by qPCR from a pool of 20 differentially expressed lncRNAs with the highest fold changes. The results of qPCR were 100% concordant microarray approach indicating that the actual number of differentially expressed lncRNAs may have been >10 in this study [29]. Of the validated results, the most interesting finding was *MEG3*, which was the most consistently downregulated lncRNA in PitNET [29,158,159]. *MEG3* has tumor suppressive characteristics, and it inhibits cell proliferation via p53 dependent and independent pathways [160]. LncRNA *SNHG1* was also reported to function as a miRNA sponge, as its expression positively correlated with tumor invasiveness [160,161]. Wang et al. investigated *SNHG1* in a PitNET context by comparing non-invasive primary tumor samples with invasive, and observed expression was positively associated with tumor invasiveness. Further in vivo and in vitro data within the study reinforced this observation and showed that *SNHG1* increased cell proliferation, migration and promoted EMT in PitNETs. This significant role in tumor progression may be explained by *SNHG1’s* ability to sponge four miRNAs with tumor-suppressive characteristics, i.e., miR-302, miR-372, miR-373, and miR-520 [162].

Another lncRNA closely associated with PitNETs is *H19*. Wu et al. identified this association by performing microarray-based lncRNA analysis in lactotroph PitNETs versus normal pituitary autopsy glands [28]. Of the 58 differentially expressed lncRNAs, *H19* was consistently downregulated in all lactotroph PitNETs samples. *H19* was further validated in 37 PitNET samples, where it was also significantly downregulated. The authors performed further in vitro and in vivo studies revealing *H19* overexpression significantly reduced tumor growth. Its direct influence on tumor growth was explained by its binding to the TOS domain of 4E-BP1, a translation repression protein. *H19* hindered 4E-BP1 interactions with the raptor protein of the mammalian target of rapamycin complex 1 (mTORC1), which stopped 4E-BP1 phosphorylation [28]. Previous studies indicated that 4E-BP1 phosphorylation led to its dissociation from the eIF4E translation factor, and uncontrolled eIF4E regulation promoted tumorigenesis [163]. These observations inferred *H19* not only served as a potential biomarker of PitNETs, but also acted as a potential therapeutic target. Another *H19* study by Zhang et al. investigated the therapeutic effects of intravenously injected exosomal *H19* into a mouse xenograft pituitary tumor model [164]. The data showed that exosomal *H19* administration not only reduced tumor growth in PitNET xenografts, but increased sensitivity to cabergoline, a common therapeutic for lactotroph PitNETs [164]. These reports indicated lncRNAs influenced PitNET development and treatment responses, therefore, more studies on lncRNA should uncover molecular mechanisms behind these therapeutic functions.

## 6. Translational Perspective

**Figure 1:**
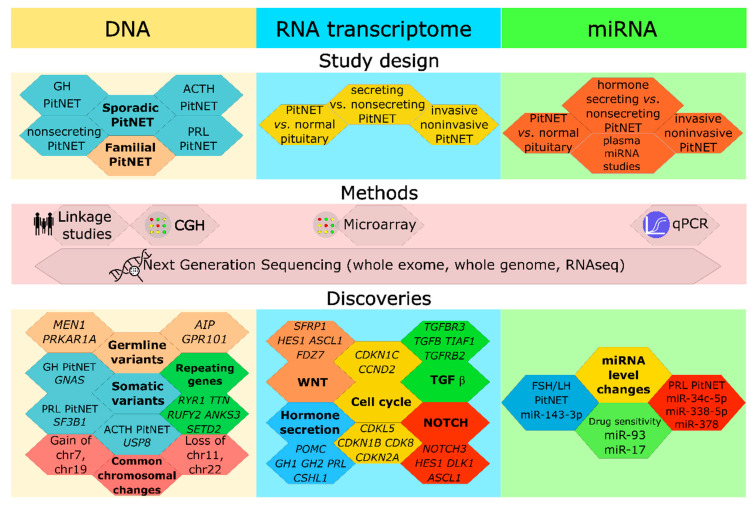
Summary of main “omics” studies in PitNETs grouped by investigated nucleic acid type. The top part shows the most popular study designs by molecule type. The middle section represents the most common methods, with next-generation sequencing having a central role in the investigation of PitNET nucleic acids nowadays. The selection of main discoveries is displayed in the lower part of the figure with the colour encoding of the nucleic acid origin, signalling pathways or related description of the relation of the discoveries to certain study design.

While several studies indicated potential candidate molecules involved in PitNET pathogenesis (Figure 1), e.g., genetic and genomic changes (*AIP*, *MEN1*, *GNAS*, and *USP8*), transcriptomic alterations (Notch, Wnt, TGF-β pathways, and cell cycle regulators), and non-coding nucleic acids (miR-34c-5p, miR-338-5p, and miR-378 tumor-suppressive miRNAs), none have proven reliable diagnostic markers or actionable targets for PitNET monitoring and management. However, it is plausible that future all-encompassing tests/assays will contain multiple targets for various PitNET subtypes. For example, a potential circulating nucleic acid based panel, encompassing cell-free DNA testing of *USP8* mutations and miR-16-5, miR-145-5p and let-7g-5p, could be used to efficiently prescribe pasireotide treatment and monitor its effectiveness in ACTH-secreting PitNETs [68,143]. Even though *USP8* somatic variants have not been detected in cell-free DNA as of yet, Megnis et al. showed that cell-free DNA of PitNET origin may be detected [165].

Interesting observations on the mutational burden of PitNET suggested that tumors with driver mutations (*GNAS* and *USP8*) had lower overall mutational rates, while tumors without drivers gradually gained more genetic alterations which ultimately promoted tumorigenesis. Therefore, the presence of driver mutations in tumor tissue indicates the cause of tumor development. To corroborate this, a recent study reported that the presence of drivers led to significantly altered transcriptome profiles in tumor tissues [25]. But direct links to transcriptome profiles in tumors are hard to envisage, as several studies indicated various related signaling pathways [24,84,86,93,108] and their functional relation to drivers needs to be further studied that could bring more actionable and translational candidates for practical application.

The heterogeneity of the involved mechanisms in PitNET pathogenesis is clear, therefore, novel methods using noninvasive approaches would be useful to overcome a major prognostic obstacle; pituitary gland accessibility for biopsy sampling. One study proposed the detection of *GNAS* mutations in patient plasma samples [165], however, the authors stressed the importance of method sensitivity thresholds. MiRNA studies indicated a tendency for the upregulation of specific miRNA subtypes in plasma, which are downregulated in PitNETs and other tumor tissues [140,143,146,147,152,154]. Unequivocally, these observations indicate specific underlying mechanisms, which downregulate miRNA subgroups in tumors to promote tumorigenesis. With the development of new more sensitive methods, and an increased knowledge of PitNET development-related markers, the prognostic and enhanced management feasibility may be more attainable in the future.

## 7. Conclusions

This review systematically summarized data from different large-scale PitNET sequencing studies and indicated potential overlapping gene candidates for the disease. Information provided here, in searchable tabular format, will provide a valuable resource for other PitNET researchers to screen overlapping markers pertaining to their own results.In conclusion, large-scale molecular studies successfully identified factors and mechanisms implicated in PitNET development. In particular, germline and somatic genome alterations (*AIP*, *MEN1*, *GNAS*, and *USP8*), dysregulated signaling pathways (Notch, Wnt, TGF-β, and cell cycle regulation), and tumor microenvironment factors were characterized, while the role of other species of RNA awaits full characterization. Our systematic review formulated the hypothesis that tumor-suppressive miRNAs were selectively downregulated in PitNET tissue, but were present at high levels in plasma, potentially allowing tumor growth, however, further functional studies are required to support this theory. So, what is next? We must understand how potential drivers in PitNETs and altered gene expression promotes tumorigenesis, and how to target these mechanisms using novel therapies and diagnostic procedures. Future translational perspective must link these findings with noninvasive marker analysis, such as miRNAs. Thus, with appropriate technological advancements, this approach could provide robust diagnostic markers and treatment management strategies for patients with PitNET.

## Figures and Tables

**Table 1:** Studies with overlapping candidate factors involved in PitNETs related to tumor growth and invasiveness.

**Publications and study design**	Kim et al. [24] 3 noninvasive non-functioning vs. 11 invasive non-functioning PitNET	Falch et al., 2018 [27] 4 fast growing vs. 4 slow growing gonadotroph PitNET	Cao et al., 2015 [98] 8 invasive vs. 8 non-invasive PitNET	Wierinckx et al., 2007 [89] 3 non-invasive vs. 4 invasive vs. 3 aggressive prolactinomas	Chen et al., 2017 [99] 3 invasive non-functioning vs. 4 invasive non-functioning PitNET	Yu et al., 2016 [97] 3 invasive non-functioning vs. 4 invasive non-functioning PitNET	De Araujo et al., 2017 [84] 4 non-invasive micro vs. 5 non-invasive macro vs. 3 invasive macro corticotroph PitNET	Galland et al., 2010 [83] 22 invasive vs. 18 non-invasive gonadotroph PitNET
Falch et al., 2018 [27] 4 fast growing vs. 4 slow growing gonadotroph PitNET	*SERPING1; AEBP1*							
Cao et al., 2015 [98] 8 invasive vs. 8 non-invasive PitNET	*FRMPD1*	*F3*						
Wierinckx et al., 2007 [89] 3 non-invasive vs. 4 invasive vs. 3 aggressive prolactinomas	no overlap	***TRIM36 ^1^***	*GAL3ST3*					
Chen et al., 2017 [99] 3 invasive vs. 4 invasive non-functioning PitNET	no overlap	no overlap	no overlap	no overlap				
Yu et al., 2016 [97] 3 invasive vs. 4 invasive non-functioning PitNET	no overlap	no overlap	*POR*	no overlap	*EZR; SLC2A1*			
De Araujo et al., 2017 [84] 4 non-invasive micro vs. 5 non-invasive macro vs. 3 invasive macro corticotroph PitNET	*TGM2*	*SLC1A2; RYR2; ZNF676*	no overlap	no overlap	no overlap	no overlap		
Galland et al., 2010 [83] 22 invasive vs. 18 non-invasive gonadotroph PitNET	no overlap	no overlap	no overlap	no overlap	no overlap	no overlap	*CD200*	
Levka et al., 2012 [85] 8 low cadherin vs. 8 high cadherin somatotroph PitNET	*SLC24A2; FAT1*	*DCLK1; **TRIM36***	no overlap	***TRIM36***	no overlap	no overlap	*VAT1L; ELAVL3; CDKN1B; SEZ6L; NIN; FXYD5; SEPT3; CCND2; SV2B*	no overlap

^1^ Highlighted in bold are factors overlapping in more than two studies.

**Table 2:** Studies with overlapping candidate factors found in PitNETs compared to normal pituitary tissues.

**Publications and study design**	Ren et al., 2018 [86] PitNET vs. normal pituitary tissue (ACTH secreting)	Evans et al., 2008 [93] PitNET vs. normal pituitary tissue (PRL secreting)	Li et al., 2017 [87] PitNET vs. normal pituitary tissue (gonadotroph secreting)	Cai et al., 2014 [94] PitNET vs. normal pituitary tissue (gonadotroph secreting)	Michaelis et al., 2011 [88] PitNET vs. normal pituitary tissue (gonadotroph secreting)	Moreno et al., 2005 [92] PitNET vs. normal pituitary tissue (nonfunctional)
Evans et al., 2008 [93] PitNET vs. normal pituitary tissue (PRL secreting)	not comparable ^1^					
Li et al., 2017 [87] PitNET vs. normal pituitary tissue (gonadotroph secreting)	not comparable	not comparable				
Cai et al., 2014 [94] PitNET vs. normal pituitary tissue (gonadotroph secreting)	not comparable	not comparable	no overlap			
Michaelis et al., 2011 [88] PitNET vs. normal pituitary tissue (gonadotroph secreting)	not comparable	not comparable	***POMC**^2^; **GH1**; **GH2**; PRL; BTG2; **GAL**;*	***F3*** *; POR; BCAT1; COL18A1;*		
Moreno et al., 2005 [92] PitNET vs. normal pituitary tissue (nonfunctional)	not comparable	not comparable	not comparable	not comparable	not comparable	
Hu et al., 2019 [95] Different types * of PitNETs vs. normal pituitary tissue * for comparison markers identified in subgroups (ACTH secreting, PRL secreting, nonfunctional) were extracted from supplementary data separately and comparison carried out only type specifically	***PMAIP1*** *; TSHB*	***POMC*** *; **GH1**; **DLK1**; **GH2**; **PON3**; **CSHL1**; **NNAT**; RBP4; IGFBP5; GPC3; **GAL**; **NPTX2**; GADD45G; CXCR4; **F3**; TGFBR3; GADD45B; IGFBP3; RPGR; CEBPD; GJA1; **CCL2**; MAFF; CDKN2A; CDKN1C; SDC4; CLDN3; POU1F1; RRAS2;*	not comparable	not comparable	not comparable	*GLCE; EPHB6; CABP1; DCX; EFNB3; CSPG5; PITX2; GNB3; GATA3; PBX3; HIST2H2BE; SPOCK3; NLGN1; IDH1; ATP1B2; ENO2; DPYSL3; KCNK3; VSNL1; FAIM2; SLC22A4; TM7SF2; SEZ6L; EPS8; FOLR1; IFI44; STC1; KCNJ6; ODC1; DUSP4; COL4A5; KDELR3; BBOX1; **NPTX2**; **NNAT**; CREM; HTATSF1; ID3; AMOT; THBS2; NR4A2; AGR2; RGS16; CEL; CGA; **PMAIP1**; KLK11; ID4; IMPA2; ID1; **CCL2**; **CSHL1**; BLM; **PON3**; PRL; **GAL**; SELL; THBS4; **DLK1**; **GH2**; **GH1**;*

^1^ “Not comparable” was assigned to the studies where different PitNET types were used and direct comparison of the discovered markers was considered inappropriate. ^2^ Highlighted in bold are factors overlapping in more than two studies.

**Table 3:** Studies on PitNET associated miRNAs.

miRNA	Expression	Sample Type	PitNET Type	Reference Sample Set	Study
miR-15a	🡫	Tumor tissue	GH, PRL	Normal pituitary tissues	Bottoni et al., 2005 [120]
miR-16-1	🡫	Tumor tissue	GH, PRL	Normal pituitary tissues	Bottoni et al., 2005 [120]
miR-34c-5p	🡫	Tumor tissue	PRL	Normal pituitary tissues	He et al., 2019 [30]
miR-338-5p	🡫	Tumor tissue	PRL	Normal pituitary tissues	He et al., 2019 [30]
miR-378	🡫	Tumor tissue	PRL	Normal pituitary tissues	He et al., 2019 [30]
miR-665	🡩	Tumor tissue	NFPA (invasive phenotype)	NFPA (non-invasive phenotype)	Zhang et al., 2019 [122]
miR-149-3p	🡫	Tumor tissue	NFPA (invasive phenotype)	NFPA (non-invasive phenotype)	Zhang et al., 2019 [122]
miR-93	🡩	Tumor tissue	PRL (bromocriptine resistant phenotype)	PRL (bromocriptine sensitive phenotype)	Wu et al., 2014 [133]
miR-17	🡩	Tumor tissue	PRL (bromocriptine resistant phenotype)	PRL (bromocriptine sensitive phenotype)	Wu et al., 2014 [133]
miR-143-3p	🡫	Plasma (after resection of PitNET)	FSH/LH	Plasma (before resection of PitNET)	Németh et al., 2019 [140]
miR-16-5p	🡩	Plasma	ACTH	Plasma of ectopic CS patients	Belaya et al., 2020 [143]
miR-145-5p	🡩	Plasma	ACTH	Plasma of ectopic CS patients	Belaya et al., 2020 [143]
let-7g-5p	🡩	Plasma	ACTH	Plasma of ectopic CS patients	Belaya et al., 2020 [143]

🡩—upregulated expression, 🡫 downregulated expression.

## Supplementary Materials

The following are available online at https://www.mdpi.com/2072-6694/13/6/1395/s1, Table S1: The list of pituitary neuroendocrine tumors candidate markers described in the review. Table S2: Large scale PitNET transcriptome studies that were used for searching for overlapping candidate factors involved in PitNET development.
